# The Full Scope: Standardization of the Use of Endoscopy for Earlier Diagnosis of Stomach Cancer in the USA

**DOI:** 10.1245/s10434-026-19765-0

**Published:** 2026-05-06

**Authors:** Gregory Sigler, Brittany Walker, Jennifer Weiss, Mark Benson, Deepak Gopal, Patrick Pfau, Kaitlyn J. Kelly

**Affiliations:** 1 Department of Surgery, University of Wisconsin Hospital & Clinics, Madison, WI USA; 2 Department of Gastroenterology and Hepatology, University of Wisconsin Hospital & Clinics, Madison, WI USA

## Abstract

**Background:**

The USA is a low-incidence country for gastric cancer and screening is not performed. The use of diagnostic endoscopy (EGD) is not well standardized. As a result, patients most commonly have advanced-stage disease at diagnosis.

**Patients and Methods:**

All patients with gastric cancer treated at a single center over the past 10 years were identified, and demographics, presenting symptoms, diagnostic evaluations, stage at diagnosis, and outcomes were analyzed.

**Results:**

A total of 249 patients with gastroesophageal junction (GEJ) or gastric or GEJ adenocarcinoma were identified. The presence of gastrointestinal (GI)-related symptoms at diagnosis was associated with more advanced disease (*p* < 0.001), but 73% of stage I patients were symptomatic. The median self-reported duration of symptoms prior to first EGD was 2 months (range 0–35 months), but nearly 50% of patients with stage IV were diagnosed by cross-sectional imaging before performance of EGD. In addition, 182 patients (73%) were participating in colon cancer screening. The median overall survival (OS) of the entire cohort was 23.0 months (95% CI 14.4–31.6) and was not reached for stage I patients, 47 months (95% CI 24.3–69.7) for stage II, 23.0 months (95% CI 19.9–26.3) for stage III, and 11.0 months (95% CI 6.6–15.4) for stage IV (*p* < 0.001). Non-EGD method of original diagnosis was an independent predictor of poorer survival, regardless of stage (HR 1.46, 95% CI 1.03–2.09;* p* = 0.036).

**Conclusions:**

Diagnostic EGD is underutilized in symptomatic patients, particularly those with dyspepsia, while most patients were participating in colon cancer screening. Novel protocols to standardize the use of diagnostic EGD should be investigated in the USA.

Gastric cancer is the fifth most common cancer and the fifth leading cause of cancer-related death worldwide. It is a disease where stage is strongly correlated with survival and where screening has definitively been shown to improve survival in high-incidence regions.^1^ The USA is a relatively low-incidence country for gastric cancer, with just over 30,000 new cases expected in 2025. The median age of onset of gastric cancer in the USA is 68 years, although recent studies have shown that earlier-onset disease is increasing.^2^ While screening for colorectal cancer with colonoscopy or stool-based DNA testing is readily performed in the USA, screening for gastric cancer is not. As a result, most patients with gastric cancer in the USA and other non-screening countries have advanced-stage disease at diagnosis.^2^

Gastric cancer staging is based on depth of tumor penetration into the wall of the stomach, involvement of regional lymph nodes, and the presence or absence of distant metastases. Patients with early stage disease can often be treated with surgical resection alone and can readily be cured. Patients with loco-regionally advanced disease require multidisciplinary treatment with chemo-immunotherapy and surgery, and only approximately 30–40% achieve 5-year survival.^3^ Patients with stage IV disease are treated with palliative intent chemo-immunotherapy and other targeted agents that are very costly, and live on the order of 1 year from diagnosis. The overall 5-year survival for all patients with gastric cancer in the USA is a dismal 38%, which is likely in large part due to late presentation and advanced stage at diagnosis.^2,3^

Anecdotally, many patients who present with locally advanced or metastatic disease endorse having had symptoms for long periods of time prior to receipt of endoscopy (EGD). They are commonly treated with a proton pump inhibitor for months prior to receipt of EGD. Indications for diagnostic EGD are not well standardized and guideline recommendations are often based on preexisting diagnoses such as gastroesophageal reflux disease (GERD) or Barrett’s esophagitis. The American Society for Gastrointestinal Endoscopy (ASGE) does make a recommendation for diagnostic EGD for any patient with new-onset dyspepsia after age 50 years, but this has been controversial, and age 60 years has been recommended for the dyspepsia indication by the American Gastroenterological Association (AGA).^4,5^ Considering that screening colonoscopy is performed in the USA for patients age 45 years and older, which involves gastroenterology consultation, there is an opportunity for selective performance of EGD. In the current study, we evaluated a comprehensive U.S. dataset of patients with gastric cancer to assess patient presentation, participation in colorectal cancer screening, and utilization of EGD across the different stages to identify opportunities for earlier gastric cancer diagnosis. We hypothesized that EGD was likely performed late following patient-reported symptom onset, that early stage patients were likely asymptomatic, and that patients with gastric cancer possibly had low compliance with colorectal cancer screening.

## Patients and Methods

### Patients and Clinical Data

This study was approved by the University of Wisconsin Institutional Review Board. All patients with gastric or gastroesophageal junction adenocarcinoma treated at the University of Wisconsin from January 2014 to January 2024 were identified by diagnostic codes (ICD-9: 151.1–151.9; ICD-10: C16.0–C16.9). Chart review was performed to collect demographic variables, presenting signs and symptoms, diagnostic evaluations, clinical stage at diagnosis, treatment related variables, and outcomes, including date of last follow-up, vital status, and date of death when applicable. Demographic and history variables included date of birth, sex, race, ethnicity, family history of cancer including type, known genetic predisposition to cancer, personal history of gastric surgery, participation in colon cancer screening, method of colon cancer screening (colonoscopy, virtual colonoscopy, multitarget stool DNA (mt-sDNA)), and any history of *Helicobacter pylori* (*H*. *pylori*) gastritis. In the analysis of patients participating in screening colonoscopy, only patients who were 45 years old or older, or those who had a known germline predisposition that warranted screening at a younger age, were included. Disease-related variables collected included date of diagnosis, method of initial diagnosis of gastric mass (EGD versus other modality, which included computed tomography (CT), magnetic resonance imaging (MRI), positron emission tomography (PET), and by intraoperative finding), patient-reported symptoms at diagnosis, self-reported duration of symptoms prior to diagnosis, presence of other findings on EGD, clinical stage at diagnosis, histologic subtype (intestinal, diffuse, or unknown), receipt of chemotherapy, duration of chemotherapy, receipt of surgery, date of last follow-up, vital status, and date of death when applicable.

The self-reported duration of symptoms was extracted from initial medical or surgical oncology consultation notes after the gastric cancer diagnosis was confirmed. Symptoms at diagnosis as reported by patients were classified as none, dyspepsia (defined as two or more of “abdominal pain/discomfort, nausea, early fullness, bloating, heartburn, and indigestion” occurring chronically or more than a solitary episode), reflux (defined as “heartburn” or “reflux” as only reported symptom), and dysphagia (defined as “difficulty swallowing” or “food sticking”). Anemia or gastrointestinal bleeding were recorded as the presenting sign/symptom when they were the indication for EGD. No patients presented with unintentional weight loss as the only initial reported symptom, but the presence of weight loss reported in the History of Present Illness or in the Review of Systems in the initial consult notes was also recorded.

### Statistical Analysis

Categorical variables were summarized as counts and percentages, and continuous variables were summarized as medians and ranges. Baseline characteristics were compared among stage groupings using the chi-squared or Fisher’s exact test for categorical variables and the Wilcoxon rank-sum test for continuous variables. Overall survival (OS) was calculated from the date of diagnosis to the date of death or date of last follow-up. Kaplan–Meier plots and log-rank tests were used to compare OS by stage and by method of diagnosis. Cox proportional hazards regression models were used to estimate hazard ratios (HRs) and 95% confidence intervals (CIs) of OS by method of diagnosis result adjusting for other variables known to be associated with survival. A two-sided *p*-value < 0.05 was considered statistically significant. All statistical analyses were performed using Statistical Package for the Social Sciences (SPSS) (version 31.0.0.0 (117)).

## Results

### Baseline Demographic and Clinical Data

A total of 249 patients with gastroesophageal junction (GEJ) or gastric adenocarcinoma were identified. The median age at diagnosis was 66 years (range 19–95 years), and 93 patients (37%) were female. A total of 216 patients (86%) were white, 12 (5%) were Asian, 13 (5%) were African American, and race was not reported for 8 (3%). Only 32 (13%) had a current or past diagnosis of *H*. *pylori* gastritis. A total of 133 patients (54%) had a family history of cancer in a first-degree relative, 10 (4%) had a known genetic predisposition placing them at high-risk for gastric cancer, and 8 (3.2%) had a history of prior gastric surgery for peptic ulcer disease or bariatric indication.

**Fig. 1:**
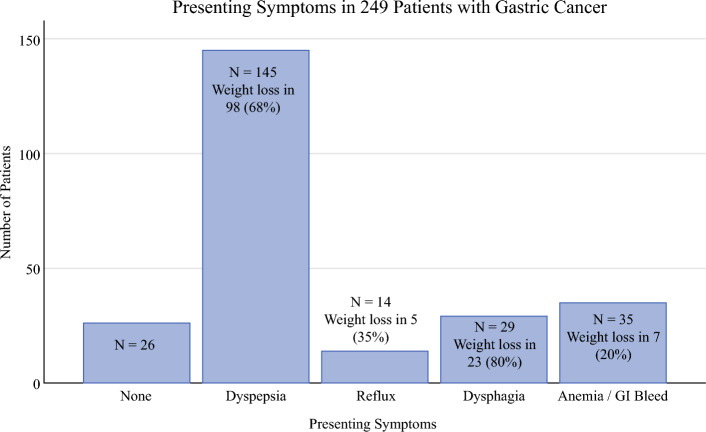
Bar graph illustrating the presenting symptoms/signs in 249 patients with gastric cancer, with number and percentage of patients with weight loss within 1 year of diagnosis in each group

In addition, 40 patients (16%) had stage I disease at diagnosis, 109 (44%) had stage II/III, and 100 (40%) had stage IV. Age was statistically significantly associated with stage at diagnosis, with stage IV patients being younger (median 61 years, range 19–95 years) than stage I (median 71 years, range 28–93 years) (Table 1). The presence of symptoms at diagnosis was statistically significantly associated with more advanced disease (*p* < 0.001), although the majority of stage I patients (73%) were symptomatic. In this cohort, dyspepsia was the most common presenting symptom, occurring in 58% of patients (Fig. 1). Symptoms differed across stages. The most common symptoms in stage I disease were anemia/gastrointestinal (GI) bleed (30%) and dyspepsia (30%). With advancing stage, dyspepsia, dysphagia, and weight loss became the most common symptoms at presentation (Table 2). Utilization of EGD and time from symptom onset to EGD also varied with presenting symptom (Table 3). Patients who presented with anemia or GI bleed, while a minority, were most likely to receive an EGD, and to receive it quickly (median of 0.5 months). Patients with reflux and dysphagia were also more likely to receive an EGD as their initial diagnostic test, but with a significant wait time (median 2.5–3.0 months) from symptom onset. Patients presenting with dyspepsia were least likely to have an EGD as their initial diagnostic test, and also had a significant delay to receiving it (Table 2). Utilization of EGD was also different across stages. EGD was the method of initial diagnosis for 85% of patients with stage I disease versus only 54% of those with stage IV disease (*p* < 0.001).

**Table 1:** Demographic and disease-related variables of 249 patients with gastric adenocarcinoma

Variable	*N* (%)
Age*	66 (19–95)
Female sex	93 (37%)
Body mass index*	27.1 (13.4–53.9)
*Race*	
Black	13 (5%)
Asian	12 (5%)
White	216 (87%)
Hawaiian/Pacific Islander	1 (< 1%)
Unknown/not reported	7 (3%)
*Ethnicity*	
Hispanic	17 (7%)
Not Hispanic	232 (93%)
Known genetic predisposition	10 (%)
Family history of any cancer	133 (54%)
Family history of gastrointestinal cancer	62 (25%)
Family history of gastroesophageal cancer	17 (7%)
Colonoscopy within last 10 years	182 (73%)
Any Cologuard test	17 (7%)
Present or past *Helicobacter pylori*	32 (13%)
Hx prior gastric surgery	8 (3%)
Other findings on EGD*	80 (32%)
*Lauren type*	
Intestinal	103 (42%)
Diffuse	73 (29%)
Unknown	73 (29%)
Stage at diagnosis	
I	40 (16%)
II/III	109 (44%)
IV	100 (40%)

^*^Included gastritis, polyps, ulceration, mucosal irregularity

**Table 2:** Symptoms at presentation in patients with gastric cancer stratified by stage

Symptoms	Stage I (%) *N* = 40	Stage II/III (%) *N* = 109	Stage IV (%) *N* = 100	*p*-Value
Any	29 (73)	101 (93)	98 (98)	< 0.001
Dyspepsia	12 (30)	63 (58)	70 (70)	< 0.001
Reflux	1 (3)	7 (6)	6 (6)	
Anemia/GI bleed	12 (30)	16 (15)	5 (5)	
Dysphagia	4 (10)	30 (28)	33 (33)	
Weight loss	7 (18)	58 (53)	70 (70)	< 0.001

**Table 3:** Method of diagnosis and time to first endoscopy in 249 patients with gastric cancer by presenting symptom

	Asymptomatic (*N* = 26)	Dyspepsia (*N* = 145)	Dysphagia (*N* = 29)	Reflux (*N* = 14)	Anemia/GI bleed (N = 35)	*p*-Value
EGD as method of first diagnosis	18 (65%)	89 (61%)	24 (83%)	12 (86%)	30 (91%)	0.003
Time from symptom onset to EGD (months)	0	2.5 (0.4–35.0)	3.0 (0–12.5)	3.0 (0.25–16.0)	0.5 (0–11.0)	< 0.001

### Participation in Colorectal Cancer Screening and Utilization of EGD

A total of 23 patients (9%) were younger than 45 years at the time of gastric cancer diagnosis and were not eligible for colorectal cancer screening; 182 of 226 patients age ≥ 45 years (80%) had a colonoscopy or some form of colon cancer screening within 10 years of their gastric cancer diagnosis (95% colonoscopy, 4% Cologuard, 1 (< 1%) virtual colonoscopy). Participation in colon cancer screening was not statistically significantly different across the gastric cancer stages (Table 4). The median age of patients not participating in colon cancer screening was 63 years and for those who were, was 67 years. The median time from colonoscopy to EGD for patients who had both tests was 12.0 months (range 0–86.0 months) for stage I patients, 31.0 months (range 0–121.0 months) for stage II/III patients, and 38.5 months (range 0–210.0 months) for stage IV patients (*p* = 0.069).

**Table 4:** Demographic, Diagnostic, and Treatment-Related Variables for Gastric Cancer Patients Stratified by Stage

Variable	Stage I *N* = 40	Stage II/III *N* = 109	Stage IV *N* = 100	*p*-Value
Age (median, years)	71 (28–93)	67 (34–88)	61 (19–95)	0.001
Body mass index	28 (19–43)	27 (17–53)	26 (13–54)	0.145
*Race*				
White	33 (83%)	96 (88%)	87 (87%)	0.619
Black	1 (2%)	5 (5%)	7 (7%)	
Asian	4 (10%)	5 (5%)	3 (3%)	
Unknown	2 (5%)	3 (2%)	3 (3%)	
*Ethnicity*				
Hispanic or Latino	2 (5%)	10 (9%)	5 (5%)	0.639
Not Hispanic or Latino	38 (95%)	99 (91%)	95 (95%)	
Family Hx of cancer	22 (55%)	56 (51%)	55 (55%)	
Colonoscopy within 10 years	33 (81%)	87 (80%)	64 (64%)	0.062
Method of diagnosis				
Endoscopy	34 (85%)	76 (70%)	54 (54%)	< 0.001
Cross-sectional imaging/other	6 (15%)	33 (30%)	46 (46%)	
Median duration of symptoms prior to first EGD* (months)	0.9 (0–12.0)	2.6 (0–35.0)	2.5 (0–76.0)	< 0.001
Median time from last colonoscopy to EGD (months)	12.0 (0–86.0)	31.0 (0–121.0)	38.5 (0–210.0)	0.069
Number of patients with colonoscopy within 1 year of gastric cancer diagnosis	16 (40%)	26 (24%)	20 (20%)	0.010
Utilization of surgical resection	37 (90%)	70 (82%)	9 (9%)	< 0.001
Utilization of systemic chemotherapy	20 (50%)	73 (85%)	88 (88%)	< 0.001
Median duration of systemic chemotherapy (months)	1.5 (0–14.0)	2.0 (0–36.0)	6.3 (0–96.2)	< 0.001
Median overall survival (months)	NR	47.0 (24.3–69.7)	11.0 (6.6–15.4)	< 0.001

^*^For patients who had an EGD

In addition, 33 patients (18%) had an EGD done at the same time as their colonoscopy (bidirectional endoscopy). Of these, the colonoscopy was done to investigate GI symptoms or new-onset anemia and was not truly a screening colonoscopy in eight patients (4%). Two of these patients were aged 46–50 years and were due for their first colonoscopy, and the remining six had been participating in screening colonoscopy previously. In the remaining 25 patients who had bidirectional endoscopy, screening colonoscopy was being performed and an EGD was added due to a specific symptoms or element of history, including: high-risk germline mutation (1), chronic GERD (4), dysphagia (6), dyspepsia (3), history of or suspected atrophic gastritis/pernicious anemia (4), history of distal gastrectomy with Billroth II anastomosis (1), suspicion of polyposis syndrome (2), history of *H*. *pylori* (1), family history of gastric cancer (1), and positive mt-sDNA with no finding on colonoscopy (2). Of note, 19 of these 25 patients (76%) had localized gastric cancer (stage I–III) and were eligible for curative intent treatment, and 4 of 9 patients (45%) who had multitarget stool DNA testing (mt-sDNA) for colon cancer screening had a positive result with negative colonoscopy, all 4 of whom had gastric cancer on EGD that was done as further workup of the result (stage I in 2, stage IV in 2).

### Treatment-Related and Outcome

**Fig. 2:**
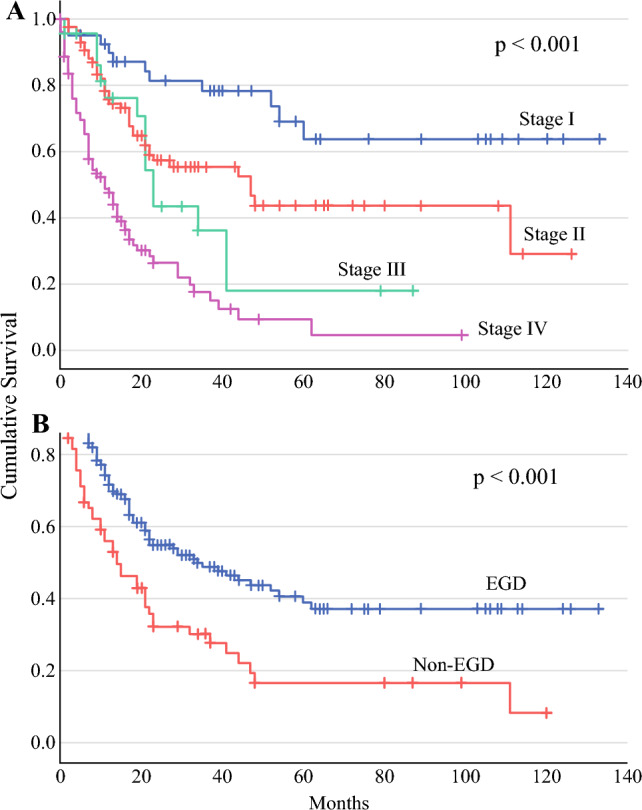
Overall survival of 249 patients with gastric cancer based on AJCC stage (**A**) and method of first gastric cancer diagnosis (**B**)

The median follow up was 38.0 months (95% CI 30.5–45.4). The median overall survival (OS) of the entire cohort was 23.0 months (95% CI 14.4–31.6) and was not reached for stage I patients. Median OS was 47 months (95% CI 24.3–69.7) for stage II, 23.0 months (95% CI 19.7–26.3) for stage III, and 11.0 months (95% CI 6.6–15.5) for stage IV (*p* < 0.001) (Fig. 2A). Aside from American Joint Committee on Cancer (AJCC) stage, method of diagnosis was also statistically significantly associated with survival on univariate analysis. Median OS of patients first diagnosed by EGD was 34.0 months (95% CI 17.6–50.4), versus 14.0 months (95% CI 6.6, 20.4) for those first diagnosed by cross-sectional imaging or other modality (Fig. 2B). Other variables associated with survival on univariate analysis included Lauren type (HR for diffuse type 1.62; 95% CI 1.09–2.41) and female sex (H.R. 1.26; 95% CI 1.06–1.49) (Table 4). When adjusting for all variables statistically significantly associated with survival, being diagnosed by EGD versus cross-sectional imaging/other, along with cancer stage, were the only independent predictors of overall survival (Table 5).

**Table 5:** Univariate and multivariate analyses of factors associated with overall survival

Variable	Univariate analysis		Multivariate analysis	
	HR (95% CI)	*p*-Value	HR (95% CI)	*p*-Value
Age	1.00 (0.98–1.01)	0.48	–	–
*Race*		0.404		
White	Ref		–	–
Black or African American	0.60 (0.24–1.46)	0.259	–	–
Asian	0.77 (0.34–1.75)	0.532	–	–
Other	0.55 (0.18–1.74)	0.309	–	–
Female sex	1.26 (1.06–1.49)	0.007	0.72 (0.51–1.01)	0.059
Lauren type		0.056		0.213
Intestinal	Ref		Ref	
Diffuse	1.62 (1.09–2.41)	0.017	1.32 (0.87–1.98)	0.196
Unknown	1.15 (0.75–1.76)	0.514	1.10 (0.72–1.68)	0.670
AJCC Stage		< 0.001		< 0.001
I	Ref		Ref	–
II	2.37 (1.21–4.64)	0.012	2.18 (1.11–4.31)	0.025
III	3.54 (1.59–7.86)	0.002	2.93 (1.30–6.60)	0.010
IV	7.31 (3.80–14.1)	< 0.001	6.17 (3.16–12.01)	< 0.001
Non-EGD method of diagnosis	1.92 (1.36–2.72)	< 0.001	1.46 (1.03–2.09)	0.036

## Discussion

Gastric cancer is routinely diagnosed late in the disease process in the USA and other Western countries where low incidence does not warrant screening. Early stage disease is often asymptomatic and screening is reserved for high-risk groups with germline predispositions such as diffuse gastric and lobular breast cancer syndrome, Lynch syndrome, Familial adenomatous polyposis, Gastric adenocarcinoma and proximal polyposis of the stomach (GAPPS) and Juvenile polyposis.^2^ This year the AGA published an expert review suggesting that additional identifiable high-risk groups exist, including first-generation immigrants from high-incidence regions and patients with family history of gastric cancer outside of documented predisposition syndrome.^6^ They recommended “considering” screening EGD in these populations. Indications for diagnostic EGD are often specific to previously documented Barrett’s esophagitis or GERD. Guideline recommendations on EGD for new onset dyspepsia are discordant and controversial, with some recommending age 50 years to start and some age 60 years.^4,5^ Dyspepsia is a general medical term that was originally meant to describe symptoms related to the upper GI tract. Varying definitions exist ranging from the presence of postprandial fullness, early satiation, epigastric pain, or epigastric burning for at least 3 months, to just epigastric pain present for at least 1 month.^4,7^ It is not a term that patients use when describing their complaints but is inferred by the provider. In practice, many consider dyspepsia synonymous with indigestion. As a result of ambiguous terminology such as “consider,” discordant guideline recommendations, and the subjectivity of the definition of dyspepsia, the use of EGD is not well standardized, neither in screening nor diagnostic settings, outside of the documented germline predisposition syndromes.

Colon cancer screening by colonoscopy and mt-sDNA testing is performed in the USA. Because colonoscopy requires GI consultation, nil per os (NPO) status, and monitored deep sedation, the addition of EGD adds minimal inconvenience or risk to the patient. Screening colonoscopy provides an opportunity for selective diagnostic EGD in individuals who are minimally symptomatic or those in a loosely defined high-risk group but without documented germline predisposition. In the current study, 76% of patients who had EGD added to a screening colonoscopy on the basis of their history, mt-sDNA result, or an upper GI-related symptom were found to have localized disease and were eligible for curative intent therapy, suggesting that questioning by providers in planning screening colonoscopy potentially elucidated the presence of upper gastrointestinal symptoms earlier than if patients had presented with those complaints independently. The data also demonstrate a hypothesis-generating finding that gastric cancer, including at stage I, can potentially be detected by mt-sDNA, while the current recommendation is that a positive mt-sDNA result without a finding on colonoscopy does not require further workup.^8^

EGD is currently the only test to reliably diagnose early stage esophagogastric cancer. EGD can also detect premalignant conditions such as atrophy, metaplasia, and dysplasia.^6^ These data are the first to show that diagnosis by EGD in patients with gastric cancer saves lives, regardless of stage. This is likely because of reduced time to tissue diagnosis and initiation of therapy in patients diagnosed by EGD as opposed to other modalities. Gastric cancer is a biologically aggressive cancer with high rates of recurrence after multidisciplinary therapy for stage II and higher disease. Stage IV disease is incurable, and the most common site of metastatic involvement is the peritoneum. Patients suffer for extended periods; often require inpatient or in home care, palliative procedures, and parenteral nutrition; and ultimately succumb to malignant bowel obstruction. Therapies for stage IV disease are expanding but are costly. The most recently approved targeted agent, zolbetuximab, when added to chemotherapy, improved survival for patients with stage IV gastric cancer with Claudin 18.2 expression by about 2.5 months over chemotherapy alone in the GLOW and SPOTLIGHT trials.^9,10^ This drug has an incremental cost-effectiveness ratio (ICER) of $273,568.01 per quality adjusted life year (QALY).^11^ Further, prompt EGD as opposed to noninvasive management strategies for new-onset dyspepsia in patients under age 50 years without alarm symptoms has been shown to be cost-effective.^12^

Countries that screen for gastric cancer achieve diagnosis at a curable stage in 60–70% of cases and currently report long-term survival of 95–99% for early stage patients and > 70% for all patients.^13^ While the disease prevalence is not high enough to warrant screening EGD for the average risk population in the USA, there is a clear need for standardization of the use of diagnostic EGD. The majority of the patients with gastric cancer reported here were participating in colon cancer screening and potentially had a missed opportunity for EGD, with nearly 50% of stage IV patients being diagnosed by cross-sectional imaging or intraoperative finding having never received an EGD. This study is limited by the retrospective design. Symptoms at diagnosis were extracted from electronic medical record history and physical notes but a standardized instrument was not utilized. Similarly, the performance of colonoscopy was in some cases at other centers long prior to referral for gastric cancer, making it impossible to determine whether patients were having any upper symptoms at the time of their colonoscopy. It is also impossible to assess clinical decision-making regarding the indications diagnostic EGD and why they were performed for symptomatic patients with stage I disease versus why they were not in the more advanced-stage symptomatic patients.

From these data, we propose a lower threshold for performance of a baseline diagnostic EGD in symptomatic patients, including in young patients. Those with dyspepsia, dysphagia, and reflux should be triaged for EGD with similar high priority as those with anemia or GI bleeding, especially when weight loss is also present. Another possible consideration to standardize the use of diagnostic EGD for patients 50 years and older would be to specifically ask them about indications for EGD while they are awaiting screening colonoscopy. This could be an area for future prospective study. If there are no pathologic or clinically significant findings on the baseline diagnostic EGD, the patient may not require further endoscopic surveillance. However, if there are, then therapy and appropriate surveillance EGD should follow. A feasible methodology to lower the threshold for and standardize the use of diagnostic EGD would allow for earlier diagnosis and potentially even prevention of gastric cancer in the US, which is a clear unmet need.
